# Comparative performance analysis of AI-based large language models in assessing cervical vertebral maturation stages on lateral cephalometric radiographs

**DOI:** 10.1186/s12903-026-08293-8

**Published:** 2026-04-16

**Authors:** Ruşen Erdem, Ahmet Yıldırım, Yavuz Selim Genç, Büşra Beşer Gül, Muhammed Enes Naralan, Orhan Cicek

**Affiliations:** 1 Department of Orthodontics, Faculty of Dentistry, Kafkas University, Kars, Türkiye; 2 Department of Orthodontics, Faculty of Dentistry, Zonguldak Bulent Ecevit University, Zonguldak, Türkiye; 3 Department of Orthodontics, Faculty of Dentistry, Giresun University, Giresun, Türkiye; 4 Department of Orthodontics, Faculty of Dentistry, Recep Tayyip Erdoğan University, Rize, Türkiye; 5 Department of Oral and Dentomaxillofacial Radiology, Faculty of Dentistry, Recep Tayyip Erdoğan University, Rize, Türkiye

**Keywords:** Cervical vertebral maturation, Artificial intelligence, Large language models, Lateral cephalogram, Orthodontics

## Abstract

**Background:**

The aim of this study was to evaluate the performance of artificial intelligence (AI)-based large language models (LLMs) in predicting cervical vertebral maturation (CVM) stages on lateral cephalometric radiographs.

**Methods:**

This retrospective study evaluated the performance of AI-based LLMs in predicting CVM stages using 120 lateral cephalometric radiographs obtained from individuals aged 6–19 years. The radiographs, which included an equal number of samples from each CVM stage, were independently classified by two experienced orthodontists, with the consensus-established stages serving as the gold standard. Five distinct LLMs (GPT-4o, GPT-o3 pro, GPT-5, GPT-5 pro, and Grok-4) were tested in separate sessions using the same command for each image. Model performance was assessed using accuracy, correlation coefficients, Bland–Altman analysis, and mean absolute error (MAE).

**Results:**

Exact-match accuracy of the AI-based LLMs ranged between 14% and 28%, while accuracy within ± 1 stage tolerance ranged from 55% to 64%. GPT-4o demonstrated the highest correlation with the reference standard (ρ = 0.616, *p* < 0.001), followed by GPT-5 pro (ρ = 0.535). Other AI-based LLMs exhibited moderate correlations (ρ = 0.3–0.4). Bland–Altman analyses indicated bias values close to zero but revealed wide limits of agreement. MAE values were comparable across AI-based LLMs, with no statistically significant differences (*p* > 0.05).

**Conclusions:**

Current LLMs did not exhibit clinically acceptable agreement with expert CVM assessments, showing wide error margins that limit their clinical utility. LLMs should presently be considered only as supportive tools. Further improvements in training and multimodal model design are needed to improve their diagnostic reliability.

## Background

In orthodontics and dentofacial orthopedics, timing is one of the key determinants of successful treatment. Growth modification largely depends on selecting the most appropriate moment for intervention, and the peak of the pubertal growth spurt is considered the optimal period to take advantage of the remaining growth potential for correcting skeletal discrepancies [1, 2]. Various biological indicators have been proposed to predict the timing of the growth spurt, including body height [3], hand–wrist development [4], dental eruption and calcification [5], menarche or voice change [6], and cervical vertebral maturation (CVM) [7].

In the past, hand–wrist radiographs were regarded as the gold standard for assessing skeletal maturity stages [8]. However, this method has the disadvantage of exposing the patient to an additional dose of radiation [9]. Cervical vertebrae visible on routinely taken lateral cephalometric radiographs can also be used to determine skeletal maturity [10]. On lateral cephalograms, CVM is evaluated based on the morphological changes of the second, third, and fourth cervical vertebrae (C2, C3, and C4) [1]. Nevertheless, the reproducibility of this method has been debated in the literature [11–13]. Consequently, the need for more reliable and standardized techniques has encouraged the integration of artificial intelligence (AI) technologies into orthodontics. The primary goal of these technologies is to assist clinicians in diagnostic and therapeutic procedures, thereby enhancing the efficiency and quality of oral health care [14].

In orthodontics, deep learning–based convolutional neural networks (CNNs) have been applied in various fields, including the prediction of growth and developmental stages, patient education, knowledge dissemination, and diagnostic and treatment planning [15]. Several studies in the literature have used CNN-based deep learning methods to evaluate CVM stages [8, 16, 17]. However, these systems require substantial computational power, advanced technical expertise, and specially curated datasets, which restrict their use mainly to professional clinical or research settings.

In recent years, large language models (LLMs) have emerged as an alternative approach. LLMs are advanced AI systems trained on large-scale datasets that enable natural language understanding and reasoning capabilities [18]. AI-based LLMs are easily accessible online and can perform a broad range of tasks [19]. Nonetheless, a comprehensive understanding of their role in orthodontic clinical practice and research remains limited. LLMs may help patients better understand their treatment plans and support orthodontists in image interpretation, diagnosis, and treatment planning. The integration of LLMs into orthodontics holds significant potential and deserves further investigation [20]. The aim of this study is to evaluate the performance of LLMs in determining CVM stages from lateral cephalometric radiographs.

## Methods

This study was approved by the Kafkas University Non-Interventional Clinical Research Ethics Committee under approval number KAÜ-TFEK 2025/08/09, dated 27 October 2025, and was conducted in accordance with the ethical principles outlined in the Declaration of Helsinki.

### Sample selection and data collection

In this retrospective study, 120 lateral cephalometric radiographs obtained for diagnostic purposes at the Department of Orthodontics, Faculty of Dentistry, Rize Recep Tayyip Erdoğan University, were analyzed. The sample consisted of individuals aged between 6 and 19 years (mean age ± standard deviation: 13.2 ± 3.1 years). The sample size was calculated using G*Power software (version 3.1.9.7, Heinrich Heine University Düsseldorf, Düsseldorf, Germany). In accordance with the study design comprising six independent groups (CVM stages 1–6), the power analysis was based on a between-groups one-way analysis of variance (one-way ANOVA) model. The effect size was determined as 0.382272 based on the between-group effect sizes reported in a comparable multimodal artificial intelligence–based orthodontic study by Arısan et al. (2025) [21]. With an alpha level (α) of 0.05 and a desired statistical power of 0.80, the minimum required sample size was calculated as 96, yielding an actual power of 0.8128891 (noncentrality parameter λ = 14.0286607; critical F = 2.3156892). In the present study, a total of 120 lateral cephalometric radiographs (20 samples per CVM stage) were included in order to further increase the statistical power of the study. Radiographs were selected from cases without any systemic or local pathology that could affect vertebral morphology, and in which the C2, C3, and C4 vertebrae were clearly visible. Radiographs with artifacts, insufficient image quality, or incomplete visualization of the vertebrae were excluded. Additionally, patients presenting with marked skeletal asymmetry or bone diseases were not included in the study. All numerical and textual information present on the radiographs was cropped to generate randomized lateral cephalometric images for analysis.

All radiographs were acquired using a Planmeca Promax 2D S2 unit (Planmeca, Helsinki, Finland) at 66 kVp, 10 mA, with an exposure time of 10.5 s and a dose area product (DAP) value of 18. During image acquisition, patients were positioned such that the Frankfurt horizontal plane was parallel to the ground. The resulting images were archived in JPEG format at a resolution of 300 dpi.

### CVM staging and reference labeling

Twenty subjects were included for each CVM stage (CVM 1–6). Each radiograph was independently evaluated by two orthodontic specialists with at least five years of clinical experience, and each image was assigned to one of the six CVM stages. Radiographs were sequentially reviewed from the department archive until 20 eligible cases meeting the inclusion criteria were identified for each of the six CVM stages. In stages where more than 20 eligible cases were available, the final 20 cases were randomly selected by the researchers to minimize selection bias. Any discrepancies between the two evaluators were resolved through consensus in a joint discussion, resulting in a single final CVM stage classification for each radiograph. These consensus-based classifications, established by two experienced orthodontists, were accepted as the “gold standard” reference stages for evaluating the performance of the AI-based LLMs in this study.

### AI-based LLM evaluations

The 120 radiographic images, divided into six groups according to CVM stages, were separately uploaded to five multimodal LLMs: four accessed via the ChatGPT platform (GPT-4o, GPT-o3 pro, GPT-5, and GPT-5 pro; OpenAI, San Francisco, USA) and Grok-4 (xAI, Austin, USA). Each LLM was prompted with the instruction:

"You are an orthodontist. This is a lateral cephalometric radiograph of a male/female patient. Please assess the skeletal maturation stage based on the Lamparski CVM method and determine the CVM stage (choose from Stage I to Stage VI)."

The evaluations were conducted in November 2025 using the following multimodal LLMs: GPT-4o (OpenAI, released May 2024, accessed via ChatGPT Plus subscription), GPT-o3 pro (OpenAI, preview version accessed November 2025), GPT-5 (OpenAI, accessed via API), GPT-5 pro (OpenAI, premium tier), and Grok-4 (xAI, accessed via grok.com subscription). All AI-based LLMs possess vision capabilities enabling direct image upload and analysis. Images were uploaded in JPEG format as exported from the imaging system at high resolution (300 dpi) with no visible artifacts in the vertebral regions.

After each evaluation, the chat history was cleared, and a new session was initiated before the next image was uploaded using the same command. All radiograph uploads and prompt submissions were performed by a single researcher in November 2025 using the same device (MacBook Air M2, 16 GB RAM; Apple, Cupertino, CA), a 4.5G internet connection, and a virtual private network (VPN) service (version 3.9; Astrill Systems Corp, Santa Clara, CA). Each radiograph was presented to each LLM only once, without any rephrasing, clarification, or repeated inquiry in cases where the model failed to respond. No additional researcher re-submitted the same queries. The obtained model outputs were recorded and stored for subsequent statistical analyses.

### Statistical analysis

The statistical analyses were performed using SPSS (version 26, IBM Corporation, New York, NY, USA) and Python (version 3.11.2; Python Software Foundation, Beaverton, OR, USA) software. The agreement between the CVM stages estimations generated by the AI-based LLMs and the gold standard CVM stages determined by expert raters was assessed using the Spearman correlation coefficient (ρ: Spearman correlation coefficient; p: statistical significance level). In addition, the mean absolute error (MAE) was calculated to quantify the average deviation between the AI-generated predictions and the reference stages. To investigate whether the differences between the AI-generated predictions and the reference stages exhibited systematic bias and whether these differences remained within acceptable limits, a Bland–Altman analysis was conducted. In the Bland–Altman plots, the differences between the predicted and reference values were plotted against the mean of each prediction-reference pair, allowing for a visual assessment of the position of each measurement pair. The analysis involved the evaluation of the mean difference (bias), the limits of agreement defined as ± 2.58 standard deviations from the mean difference, and the regression relationship between the differences and the means. A *p* < 0.05 was considered statistically significant.

## Results

The study sample comprised 120 patients (mean age 13.2 ± 3.1 years). Gender distribution was balanced (60 females and 60 males) and equal within each cervical vertebral maturation (CVM) stages (Table 1).

**Table 1:** Demographic characteristics and distribution of CVM stages in the study sample

Characteristic	*n*
Total patients	120
Female	60
Male	60
Age (years, mean ± SD)	13.2 ± 3.1
CVM Stage 1	20 (10 female / 10 male)
CVM Stage 2	20 (10 female / 10 male)
CVM Stage 3	20 (10 female / 10 male)
CVM Stage 4	20 (10 female / 10 male)
CVM Stage 5	20 (10 female / 10 male)
CVM Stage 6	20 (10 female / 10 male)

Spearman correlation analysis revealed a significant positive correlation between the reference CVM stages and all AI-based LLMs (*p* < 0.001). Among the tested AI-based LLMs, GPT-4o exhibited the highest correlation with the reference data (ρ = 0.616), followed by GPT-5 pro (ρ = 0.535) and GPT-5 (ρ = 0.363). The correlations of GPT-o3 pro (ρ = 0.319) and Grok-4 (ρ = 0.347) with the reference were comparatively weaker but remained statistically significant. Inter-model correlations indicated moderate associations between GPT-4o and other AI-based LLMs (ρ = 0.229–0.258, *p* < 0.05), whereas Grok-4 demonstrated minimal correlation with GPT-5 (ρ = 0.045, *p* = 0.626) and only a weak, nonsignificant association with GPT-5 pro (ρ = 0.152, *p* = 0.097) (Table 2).

**Table 2:** Correlation analysis between reference data and LLMs in CVM stages

Groups		References	GPT-4o	GPT-o3 pro	Grok-4	GPT-5	GPT-5 pro
References	Spearman’s rho	1	0.616	0.319	0.347	0.363	0.535
	*p*		< 0.001 ^*^	< 0.001 ^*^	< 0.001 ^*^	< 0.001 ^*^	< 0.001 ^*^
GPT-4o	Spearman’s rho		1	0.242	0.258	0.248	0.229
	*p*			0.008 ^*^	0.005 ^*^	0.006 ^*^	0.012 ^*^
GPT-o3 pro	Spearman’s rho			1	0.103	0.259	0.238
	*p*				0.261	0.004 ^*^	0.009 ^*^
Grok-4	Spearman’s rho				1	0.045	0.152
	*p*					0.626	0.097
GPT-5	Spearman’s rho					1	0.239
	*p*						0.09 ^*^
GPT-5 pro	Spearman’s rho						1
	*p*						

*p*:* p*-value, ^*^: *p* < 0.05

The accuracy of CVM stages prediction varied across LLMs depending on the tolerance level applied. When exact matches were considered, GPT-4o demonstrated the highest accuracy (28.33%), followed by GPT-5 (23.33%), GPT-5 pro (23.33%), and GPT-o3 pro (23.33%), whereas Grok-4 exhibited the lowest accuracy (14.16%). When a deviation of ± 1 stage was tolerated, overall accuracy markedly increased for all AI-based LLMs. GPT-5 pro achieved the best performance under this condition (64.16%), closely followed by GPT-4o (62.50%) and GPT-5 (60.00%). GPT-o3 pro and Grok-4 each reached an accuracy of 55.00% (Table 3) [22].

**Table 3:** Accuracy of LLMs in CVM stages prediction on cephalometric radiographs across exact and ± 1 tolerances

	GPT-4o	GPT-o3 pro	Grok-4	GPT-5	GPT-5 pro
Exact Match	28.33	23.33	14.16	23.33	23.33
Within ± 1 stage deviation	62.5	55	55	60	64.16

Numerical values are expressed as percentages

**Fig. 1:**
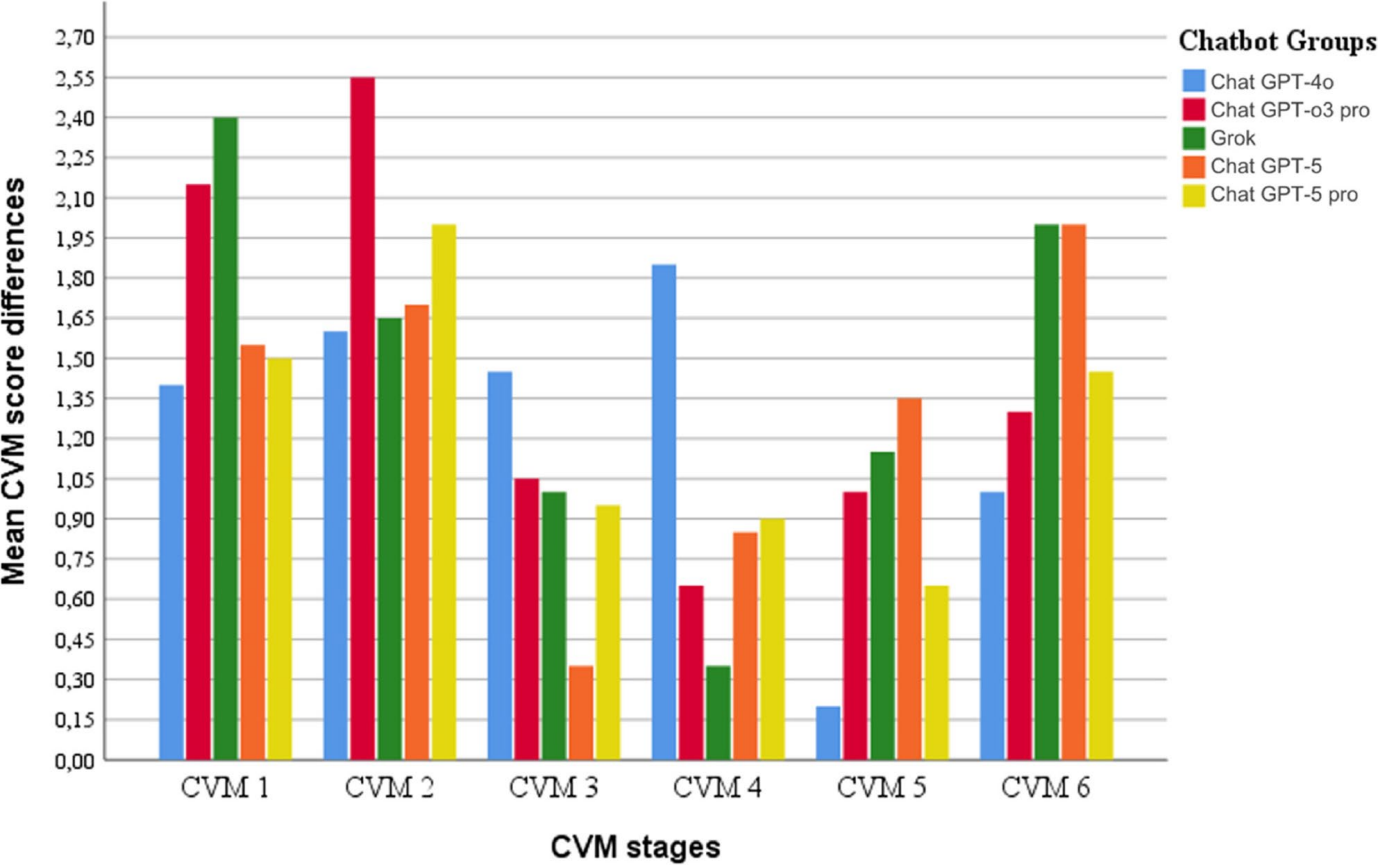
Clustered bar chart showing mean score differences of CVM stages across LLMs

Figure 1 illustrates the mean differences between the predicted and reference CVM stages across LLMs for each maturation stage. The largest deviations were observed at the early (CVM 1–2) and late (CVM 6) stages, indicating greater prediction variability during the initial and final growth phases. Among all AI-based LLMs, GPT-o3 pro exhibited the highest mean difference, particularly at CVM 2, whereas GPT-4o and GPT-5 pro demonstrated relatively smaller deviations across most stages. The mid-stage levels (CVM 3–4) showed the lowest error rates, suggesting more consistent performance of all AI-based LLMs during the intermediate maturation stages.

**Fig. 2:**
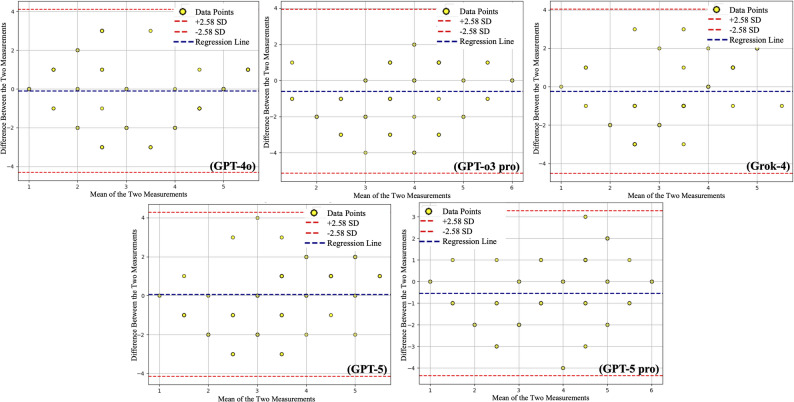
Bland–Altman plots illustrating the agreement between the CVM stage predictions and the reference data

Figure 2 shows Bland–Altman plots comparing CVM stage predictions by the LLMs with the reference data. The majority of data points for all AI-based LLMs were within the 99% confidence limits (± 2.58 SD). The mean biases were close to zero for GPT-4o, GPT-o3 pro, Grok-4, GPT-5, and GPT-5 pro. The limits of agreement ranged approximately from − 4 to + 4 across AI-based LLMs. The slopes of the regression lines, indicated by the blue dashed lines, were not statistically significant (*p* = 0.387).

The performance differences of the five LLMs were compared with the reference stages in terms of MAE values. The mean performance deviation was found to be 1.25 (SD = 1.05) for GPT-4o, 1.45 (SD = 1.16) for GPT-o3 pro, 1.43 (SD = 0.88) for Grok-4, 1.30 (SD = 0.98) for GPT-5, and 1.24 (SD = 0.97) for GPT-5 pro. According to the Bonferroni-adjusted repeated measures ANOVA test (or Friedman test), no statistically significant difference was observed among the AI-based LLMs in terms of their mean MAE values (*p* = 0.271 for ANOVA / *p* = 0.158 for Friedman).

## Discussion

The integration of LLMs into medical applications is regarded as a milestone in the expansion of AI–based approaches within healthcare [23]. In recent years, the reliability and accuracy of LLMs have begun to be investigated, particularly in clinical decision support systems, radiographic analysis, and dental applications [22, 24–26]. In this regard, LLMs have been investigated for their ability to predict bone age and growth-development stages based on hand–wrist radiographs, and varying levels of success have been reported [22]. The non-random performance demonstrated by LLMs when processing visual inputs from hand–wrist radiographs highlights the potential value of investigating their performance in estimating CVM stages using lateral cephalometric radiographs. In this context, the present study analyzed the capability of AI–based LLMs to assess CVM stages using lateral cephalometric radiographs.

The majority of studies reported in the literature are based on CNN–based deep learning architectures, which, unlike the present research, do not employ LLMs [27–30]. Therefore, the originality of the present study lies in demonstrating the applicability of AI-based LLMs for assessing CVM stages, as opposed to image-centered CNN approaches. For instance, Seo et al. (2021) compared six different CNN architectures using 600 lateral cephalograms and reported accuracies exceeding 90% for all AI-based LLMs, with the highest performance achieved by the Inception-ResNet-v2 model [27]. Li et al. (2023) developed the psc-CVM system using more than 10,000 cephalograms and obtained a weighted κ = 0.844 and ICC = 0.946, indicating very strong agreement with expert evaluators [28]. Similarly, Mohammad-Rahimi et al. (2022) showed that transfer-learning-based CNN models achieved validation and test accuracies of 62.63% and 61.62%, respectively, in six-class CVM classification tasks [29]. Li et al. also reported that CNN models provide an efficient, rapid, and reliable approach for CVM analysis and may be utilized for the development of future automated diagnostic support systems [30]. Overall, the literature confirms that CNN-based approaches can, to some extent, replicate expert CVM assessments. By contrast, the performance achieved by the LLMs in the present study was substantially lower. However, this comparison with CNN-based systems should be interpreted cautiously, as it is indirect and based on previously published studies using different datasets and methodologies rather than direct head to head comparisons on the same dataset used in the present study. 

In the present study, the accuracy of AI–based LLMs in assessing CVM stages from lateral cephalometric radiographs was evaluated by comparing their correlations with reference data. Correlation analyses between the model predictions and the reference measurements revealed statistically significant linear relationships for all AI-based LLMs (*p* < 0.001), indicating non-random results; however, high levels of correlation were not achieved. Even the GPT-4o model, which demonstrated the best performance among the AI-based LLMs, achieved only a moderate correlation with the reference values (ρ = 0.616) [31]. Although all AI-based LLMs showed statistically significant correlations with the reference standard, it is important to emphasize that correlation measures linear association rather than agreement. Statistically significant correlation does not necessarily imply clinically acceptable agreement or diagnostic reliability, as evidenced by the wide limits of agreement observed in the Bland–Altman analyses.

The accuracy levels of AI-based LLMs in correctly predicting CVM stages with exact agreement were found to be considerably low. Although accuracy rates improved when a tolerance of ± 1 stage was applied, these values still did not reach clinically acceptable levels.

The findings revealed that the reliability of LLMs varied across different CVM stages. Notably, greater scoring discrepancies were observed at CVM 1–2 and CVM 6 (Fig. 1). Although McNamara and Franchi [32] reported that CVM 6 is typically the most challenging stage to identify due to the need for careful measurement of the posterior–inferior dimensional relationship of C3 and C4, our study found the highest discrepancies at CVM 1, CVM 2, and CVM 6. This suggests that LLM models may struggle not only with the morphological characteristics of late maturation but also with the subtle anatomical features that define the early stages. As noted additionally by McNamara and Franchi [32], spike-like bony formations can appear along the inferior borders of C2–C4, mimicking an extension of the inferior border and creating the erroneous impression of early concavity or notching. These structures are, in fact, isolated islands of bone that are not connected to the vertebral bodies and may therefore contribute to misclassification. Thus, while CVM 6 remains inherently difficult to identify, the minimal morphological changes characteristic of the early stages combined with such anatomical variations may also negatively affect LLM performance.

Based on this inference, Bland–Altman analyses were conducted to determine whether model performance varied according to the progression of stages. The Bland–Altman plots visualized the agreement between the LLMs and the reference data [33]. According to the Bland–Altman analyses, the absence of a significant slope in the regression line indicates that no systematic bias was present and that the prediction errors of the AI-based LLMs were independent of the stage levels. Although the Bland–Altman plots revealed no evident systematic bias between the model predictions and the reference measurements, the wide limits of agreement (± 2.58 SD) indicate a considerable dispersion of the differences. This finding indicates that the model predictions may exhibit substantial deviations from the reference measurements, thereby suggesting that the AI-based LLMs do not demonstrate clinically consistent predictive performance.

Although the AI-based LLMs demonstrated certain levels of correlation with the reference values, indicating that their predictions were not purely random, this alone cannot be regarded as sufficient evidence of reliability. The wide limits of agreement observed in the Bland–Altman analyses do not support the partial success suggested by the correlation results. Furthermore, the low rate of exact score matches even when a tolerance of ± 1 stage was allowed, which in a six-stage classification system corresponds to considering two or three possible predictions as correct shows that the AI-based LLMs still failed to reach a satisfactory level of performance, making it difficult to characterize them as successful.

Studies focusing on the adaptation of AI models to specific professional domains indicate that fine-tuning and task-oriented training strategies can meaningfully enhance model performance. In a study comparing the base and fine-tuned versions of GPT-4, fine-tuning with domain-specific data was reported to markedly increase model accuracy [34]. In parallel, a self-supervised deep learning framework developed for periapical radiograph segmentation demonstrated that pretraining a Vision Transformer on a large set of unlabeled images, followed by fine-tuning with limited annotated data, yielded higher performance compared with fully supervised methods [35]. When considered together, these two studies show that task-specific training contributes substantially to improving the accuracy and clinical applicability of both LLMs and vision-based models. In this context, domain-adapted fine-tuning strategies may similarly provide performance improvements in future LLM based AI applications aimed at CVM analysis.

Perinetti et al. [10], demonstrated that the CVM assessment method can achieve satisfactory reproducibility when evaluators receive specific training in stage identification. A similar explanation may be applicable to the performance of AI-based LLMs. In this context, while human clinicians may exhibit “experience-related” errors, the performance of LLMs is likely influenced by “training and contextual comprehension limitations.” Accordingly, although the current limitations of AI-based LLMs render them insufficient for clinical use in CVM staging at this stage, it should be acknowledged that these systems remain open to further development and improvement.

A major limitation of this study lies in the fact that LLMs are not directly optimized for image processing, and their performance largely depends on training and contextual comprehension capacities. In addition, the use of radiographs obtained from a single center limits sample diversity and generalizability. Each radiograph was submitted to the AI-based LLMs only once using a fixed prompt, without repeated queries or prompt variations. This design choice precludes evaluation of intra-model response variability and sensitivity to prompt phrasing, potentially limiting insight into the AI-based LLMs’ robustness and optimal performance. Additionally, images were exported and uploaded in JPEG format, which involves lossy compression. Although high-resolution exports (300 dpi) were used and no visible artifacts were present in the vertebral regions, minor compression-related degradation cannot be entirely ruled out. Furthermore, the gold standard relied on consensus between only two orthodontists without assessing evaluator repeatability, potentially introducing some subjectivity despite their extensive clinical experience. These limitations should be considered when interpreting the findings, and future studies should address them using larger, multicenter datasets and optimized model training. In this context, given the rapid advancement of LLMs, the primary objective should be to first reach, and subsequently surpass, the performance levels of CNN-based models trained on large, labeled image datasets [23].

## Conclusion

This study evaluated the performance of AI-based LLMs in determining CVM stages on lateral cephalometric radiographs. Although all AI-based LLMs demonstrated statistically significant correlations with the expert consensus reference standard, their accuracy levels were insufficient for clinical implementation. In addition, the wide limits of agreement observed across all AI-based LLMs indicated considerable individual variability.

Overall, the findings suggest that current AI-based LLMs systems are not yet capable of replacing expert assessment in skeletal maturity evaluation and should presently be regarded only as supportive tools. Further improvements in visual interpretation capabilities, domain-specific fine-tuning, and training on larger orthodontic datasets are required to enhance their diagnostic reliability. Nevertheless, given the rapid pace of technological advancement, future versions of these AI-based LLMs are expected to hold meaningful potential as auxiliary diagnostic tools in orthodontic practice.

## Data Availability

The datasets used and/or analyzed during the current study are available from the corresponding author on reasonable request.

## Abbreviations

AI: Artificial Intelligence
LLMs: Large Language Models
CVM: Cervical Vertebral Maturation
CNNs: Convolutional Neural Networks
MAE: Mean Absolute Error
ANOVA: Analysis of Variance
SD: Standard Deviation
DAP: Dose Area Product
VPN: Virtual Private Network
ICC: Intraclass Correlation Coefficient
κ: Kappa coefficient
ρ: Spearman correlation coefficient
p: *p*-value
